# Thinking Styles and Regret in Physicians

**DOI:** 10.1371/journal.pone.0134038

**Published:** 2015-08-04

**Authors:** Mia Djulbegovic, Jason Beckstead, Shira Elqayam, Tea Reljic, Ambuj Kumar, Charles Paidas, Benjamin Djulbegovic

**Affiliations:** 1 University of South Florida, Morsani College of Medicine, Tampa, Florida, United States of America; 2 Program for Comparative Effectiveness Research, University of South Florida, Tampa, Florida, United States of America; 3 Division of Evidence-Based Medicine, Department of Medicine, University of South Florida, Tampa, Florida, United States of America; 4 College of Nursing, University of South Florida, Tampa, Florida, United States of America; 5 School of Applied Social Sciences, De Montfort University, Leicester, United Kingdom; 6 Department of Hematology and Health Outcomes Research, H. Lee Moffitt Cancer Center & Research Institute, Tampa, Florida, United States of America; 7 Tampa General Hospital, Tampa, Florida, United States of America; Catholic University of Sacro Cuore, ITALY

## Abstract

**Background:**

Decision-making relies on both analytical and emotional thinking. Cognitive reasoning styles (e.g. maximizing and satisficing tendencies) heavily influence analytical processes, while affective processes are often dependent on regret. The relationship between regret and cognitive reasoning styles has not been well studied in physicians, and is the focus of this paper.

**Methods:**

A regret questionnaire and 6 scales measuring individual differences in cognitive styles (maximizing-satisficing tendencies; analytical vs. intuitive reasoning; need for cognition; intolerance toward ambiguity; objectivism; and cognitive reflection) were administered through a web-based survey to physicians of the University of South Florida. Bonferroni’s adjustment was applied to the overall correlation analysis. The correlation analysis was also performed without Bonferroni’s correction, given the strong theoretical rationale indicating the need for a separate hypothesis. We also conducted a multivariate regression analysis to identify the unique influence of predictors on regret.

**Results:**

165 trainees and 56 attending physicians (age range 25 to 69) participated in the survey. After bivariate analysis we found that maximizing tendency positively correlated with regret with respect to both decision difficulty (r=0.673; p<0.001) and alternate search strategy (r=0.239; p=0.002). When Bonferroni’s correction was not applied, we also found a negative relationship between satisficing tendency and regret (r=-0.156; p=0.021). In trainees, but not faculty, regret negatively correlated with rational-analytical thinking (r=-0.422; p<0.001), need for cognition (r=-0.340; p<0.001), and objectivism (r=-0.309; p=0.003) and positively correlated with ambiguity intolerance (r=0.285; p=0.012). However, after conducting a multivariate regression analysis, we found that regret was positively associated with maximizing only with respect to decision difficulty (r=0.791; p<0.001), while it was negatively associated with satisficing (r=-0.257; p=0.020) and objectivism (r=-0.267; p=0.034). We found no statistically significant relationship between regret and overall accuracy on conditional inferential tasks.

**Conclusion:**

Regret in physicians is strongly associated with their tendency to maximize; i.e. the tendency to consider more choices among abundant options leads to more regret. However, physicians who exhibit satisficing tendency – the inclination to accept a “good enough” solution – feel less regret. Our observation that objectivism is a negative predictor of regret indicates that the tendency to seek and use empirical data in decision-making leads to less regret. Therefore, promotion of evidence-based reasoning may lead to lower regret.

## Introduction

Both emotions and analytical processes characterize decision-making [1]. Although some authors have forcibly advocated for a better understanding of emotions as determinants of our behavior and decision-making [2], the relationship between the affective and analytical components of decision-making has not been well studied, particularly in the medical field. Among the basic emotions such as fear, love, anxiety, etc., regret is particularly appealing because the decision maker feels it at both the cognitive and emotional level [1]. In fact, regret is defined as a cognitive emotion that involves counterfactual thinking [1], which means that the invocation of regret relies on considerations of alternative thinking. Such “what if” scenarios may help a decision-maker assess all possible options, where some alternative choices can be more or less regretted than others [1]. We [3–5] and others [6] have shown how regret plays a role in clinical problem solving and decision-making, which occurs by a decision-maker’s tendency to minimize or avoid regret. Similarly, Colombo et al. [7] investigated how awareness of one’s own cognitive style affects both the decision making process and the propensity to feel regret. The authors demonstrated that those prone toward intuitive reasoning might feel less regret than those who are inclined to think analytically. This study, as well as theoretical considerations, lends support to the idea that regret may tap into or relate to other cognitive processes. People prone to feel regret tend to consider more alternative solutions to a problem [3,8,9], which is characteristic of maximizing tendency during problem-solving [3,10–13].

Many, if not most, medical decisions are “high stakes”; as a result, they are colored with emotions and the desire to avoid regret by leaving “no stone unturned” in order to arrive at the best possible decision. It, therefore, makes sense to assume a relationship between regret and maximizing tendency. It has been proposed that regret can be considered as a link between the affective and deliberative aspects of decision-making and problem solving processes [10], particularly those that characterize the field of medical decision-making [3]. However, it is not clear how regret relates to other cognitive styles such as satisficing (the tendency to accept a “good enough” solution, rather than search for the “optimal” one), or the tendency to rely on intuitive, as opposed to analytical, thinking [1,14].

We, therefore, set out to assess 1) How does regret correlate with maximizing vs. satisficing in physicians? 2) How does regret correlate with other scales that measure cognitive styles (e.g. propensity towards intuitive-experiential vs. analytical-rational thinking)? 3) How does regret correlate with logical and inferential tasks? 4) How does regret vary among different medical specialties (e.g. cognitive v. procedural disciplines).

## Methods

All residents, fellows and attending physicians affiliated with the University of South Florida, Morsani College of Medicine were invited to participate in a study via e-mail. The web-based survey was comprised of questions on participant demographics (i.e. age, level of training, gender, medical specialty), individual differences among decision-making (based on well-validated scales measuring six key constructs, detailed below), and conditional inferences. The complete survey can be found in S1 Appendix. Before proceeding to the survey, all participants had to provide informed consent. The survey was approved by the USF International Review Board (No. 9047), and was administered using Qualtrics survey software. The results from analyzing differences in cognitive styles among physicians were published previously [3]. This updated paper focuses on the correlation between these various cognitive styles and regret proneness.

We administered the following scales to measure individual differences in cognitive styles:

**Maximizing Inventory**, which refers to effortful reasoning. It is comprised of three separate scales consisting of 34 items [15]:

1. The Alternative Search Scale (AS), which assesses the tendency to expend resources in exploring all possible opportunities and is directly related to maximizing. In our data it had a Cronbach’s alpha of 0.879.
2. The Decision Difficulty Scale (DD), which represents the degree of difficulty experienced when making choices among abundant options, and is also directly related to maximizing. In our data it had a Cronbach’s alpha of 0.858.
3. The Satisficing Scale, an independent construct that is not mutually exclusive from maximizing (i.e. individuals can use both strategies). In our data it had a Cronbach’s alpha of 0.746.

These scales are referred to as Maximizing-Satisficing Scales, and refer to the amount of effort that an individual is willing to expend to arrive at a solution to a problem. Maximizers will search for alternatives until they arrive at an “optimal” solution, whereas satisficers will search until they arrive at a “good enough” solution.

**Rational-Experiential Inventory** is based on the dual process theory of cognition, whereby rational v. intuitive reasoning are two distinguishable cognitive styles. The inventory consists of two item subscales consisting of 40 items that measure intuitive-experiential and analytical-rational thinking based on cognitive-experiential self-theory [16]. In our data it had Cronbach’s alpha of 0.893 and 0.888, respectively.

**Intolerance of Ambiguity Scale** measures the decision maker’s tolerance of uncertainty, i.e. the individual’s ability to feel comfortable and accept situations where variables, alternative or outcomes are poorly defined, uncertain or unclear [17]. The scale consists of 16 items; in our data it had a Cronbach’s alpha of 0.643.

**Need for Cognition** refers to the degree to which individuals prefer to engage in, and derive enjoyment from cognitive activities [18]. The scale consists of 17 items; in our data it had a Cronbach’s alpha of 0.894.

**Objectivism** refers to the tendency to seek empirical information under conditions of uncertainty and to attempt to process it in a rational and logical fashion. The objectivism scale measures the tendency to base one’s judgments and beliefs on empirical information and rational considerations [19]. It consists of 11 items; in our data the scale had a Cronbach’s alpha of 0.717.

**Cognitive Reflection Task** refers to the ability or disposition to suppress intuitive and spontaneous answers in favor of more reflective and deliberative responses [20]. The scale consists of 3 items, which are too few to test reliability [21], and explains why in our data it had a Cronbach’s alpha was only 0.599.

### Scale Reliabilities

Overall, we found that psychometric properties in the medical setting are similar to the original reports and, therefore, are satisfactory for the use in medical settings as well [3].

### Regret

There are many ways to measure regret, and various scales have been developed to dissect the role of regret related to intentions and prospective behavior [22]. However, regardless of how regret is elicited, it appears that any instrument will reliably tap into the regret construct [22]. We, therefore, measured regret by using a simple questionnaire on 1–6 point Likert scale where “1” indicates the least possible regret, and “6” the maximum possible regret people can feel (see S1 Appendix for full survey). A single scale (ranging from zero regret to 100% regret) was also used by Sorum et al [6], as well as in our previous research [4,5,23]. Both our experience and the experience by Sorum and colleagues[6] showed that this assessment of asking people to quantify the regret they feel when making choices mirrors what people themselves consider regret, and is therefore easily comprehended by study participants. These easy-to-use and easy to understand, time-efficient scales are ideal for clinical use in busy physicians.

### Assessment of Accuracy of Inferences

According to the dual processing theory of cognition, decision makers have the tendency to draw deductive inferences based on prior beliefs, regardless of logical validity. In other words, people will exhibit a “belief bias” if an argument is believable, whether or not it is logically valid. Belief bias was assessed in our study using conditional inference task with medically-relevant contents, pretested and developed for this study (see S1 Table) [3,24]

### Statistical Analysis

The characteristics of the participants were summarized using descriptive statistics. We conducted both bivariate and multivariate regression analysis.

Pearson’s correlation statistics were used to analyze how regret varies with age, as well as to analyze the relationship between regret and three pre-defined subgroups: 1) level of expertise (trainees vs. attending physicians) 2) gender (male vs. female) and 3) specialty (cognitive vs. procedural disciplines). The latter subgroup is pertinent to clinical disciplines, as the cognitive skills employed by specialists such as internists, family physicians, psychiatrists, and pediatricians are considered to differ from the problem-solving and decision-making strategies employed by surgeons. The former rely more extensively on history taking and the integration of laboratory and other clinical data to formulate optimal management strategies (i.e. “cognitive” specialties). Physicians in surgical disciplines are, however, are mostly concerned with decisions and outcomes related to surgery (i.e. “procedural” specialties) [3,25].

To protect from alpha error, we applied Bonferroni’s adjustment to correct multiple comparisons for *P* values. However, because previous studies [7,11] in the general population suggest that maximizers feel more regret than satisficers, we also tested the relationship between regret and maximizing-satisficing tendencies without Bonferroni’s correction. To identify the unique influence of the various predictors of regret, we also performed a multivariate linear regression analysis, where regret was the dependent variable, and all cognitive styles and demographic features (age, gender, training level, and discipline) were independent variables.

The differences in responses between trainees and attending physicians on the conditional inference tasks were compared with a Chi-square test. Statistical programs SPSS and Stata were used to perform all analyses.

## Results

165 trainees and 56 attending physicians (median age 31 [range 25 to 69 years]) responded to the electronic survey and were included in the study (see Table 1). As discussed earlier, all six constructs (maximizing inventory, rational-experiential inventory, intolerance of ambiguity scale, need for cognition, objectivism, and cognitive reflection tasks) showed acceptable psychometric properties.

**Table 1:** Characteristics of Study Participants.

Median Age (range)	31 (25 to 69)
Variable	N (%)
**Training Status**	
Trainees (resident/fellow)	165 (75)
Faculty (attending)	56 (25)
**Gender**	
Male	120 (54)
Female	101 (46)
**Discipline**	
Internal Medicine	37 (17)
Pediatrics	29 (13)
Surgery	19 (9)
Obstetrics and Gynecology	15 (7)
Radiology	15 (7)
Ophthalmology	12 (5)
Psychiatry	12 (5)
Other	82 (37)
**Discipline Type**	
Surgical	57 (26)
Non-Surgical	164 (74)

### Bivariate analysis: correlation between regret and other scales

Maximizing tendency, measured by both decision difficulty and alternative search, positively correlated with regret (DD: r = 0.673; p<0.001) (AS: r = 0.239; p = 0.002). Satisficing tendency, however, did not demonstrate any statistically significant correlation with regret (r = -0.156; p = 0.123). When the correlations were analyzed without Bonferroni’s correction, we observed a statistically significant negative correlation between regret proneness and satisficing tendency (r = -0.156; p = 0.021).

Interestingly, among physicians (both trainees and attending physicians), regret negatively correlated with rational, analytical thinking (r = -0.367; p<0.001), need for cognition (r = -0.282; p = 0.001), and objectivism (r = -0.230; p = 0.025). Similarly, regret was positively associated with ambiguity intolerance (r = 0.236; p = 0.019). There was no relationship between regret and intuitive-experiential thinking (r = -0.142; p = 1.00), or between regret and cognitive reflection (r = -0.006; p = 1.00).

We found no statistically significant relationship between regret and accuracy in any of the four types of conditional inferences (modus ponens: r = -0.147; p = 0.810; (denial of antecedent: r = 0.052; p = 1.00; affirmation of the consequent: r = 0.105; p = 1.00;modus tollens: r = -0.023; p = 1.00).

### Bivariate analysis: Subgroup analysis

A subgroup analysis was performed to determine whether the above results were representative of the whole study population, or if they relate to a specific subgroup of participants.

#### Age

Participants’ age ranged from 25 to 69 years, with a median age of 31. Regret proneness did not demonstrate any statistically significant relationship with age (r = -0.131; p = 0.161).

#### Level of training

165 participants were trainees (residents or fellows), and 56 were attending physicians. There was no relationship between regret proneness and satisficing tendency among trainees (r = -0.147; p = 1.00) and faculty (r = -0.220; p = 1.00). A positive relationship was observed between decision difficulty and regret among both trainees (r = 0.666; p<0.001) and faculty (r = 0.770; p<0.001). However, there was no relationship between alternate search and regret among both trainees (r = 0.208; p = 0.411) and faculty (r = 0.292; p = 1.00). Interestingly, trainees demonstrated a significant negative correlation between regret and rational-analytical thinking (r = -0.422; p<0.001), however there was no such relationship among faculty (r = -0.073; p = 1.00). The difference between these two correlation coefficients was significant (p = 0.017). There was no relationship between intuitive-experiential thinking and regret among both trainees (r = -0.123; p = 1.00) and faculty (r = -0.260; p = 1.00). There was a significant negative association between need for cognition and regret among trainees (r = -0.340; p<0.001), however not among faculty (r = -0.015; p = 1.00); the difference between the two correlation coefficients was significant (p = 0.032). Trainees showed a negative correlation between objectivism and regret (r = -0.309; p = 0.003), however faculty did not (r = 0.120; p = 1.00); the difference between these two correlations was highly significant (p = 0.005). Ambiguity intolerance was associated with regret among trainees (r = -0.285; p = 0.012), but not among faculty (r = -0.025; p = 1.00), and the difference between them was also significant (p = 0.044). We observed no relationship between regret and the cognitive reflection task among trainees (r = 0.005; p = 1.00) and faculty (r = 0.003; p = 1.00).

#### Gender

120 study participants were male, and 101 were female. There was no statistically significant correlation between regret and satisficing among male participants (r = -0.081; p = 1.00) and females (r = -0.254; p = 0.574). Within the maximizing inventory, there was a strong positive relationship between regret and decision difficulty among both males (r = 0.690; p<0.001) and females (r = 0.665; p<0.001), although there was no relationship between regret and alternate search among both males (r = 0.205; p = 1.00) and females (r = 0.278; p = 0.271). There was a strong negative relationship between regret and rational-analytical thinking among both males (r = -0.386; p<0.001) and females (r = -0.358; p = 0.013). There was no association between regret and intuitive-experiential thinking among males (r = -0.101; p = 1.00) and females (r = -0.174; p = 1.00). Males showed a negative correlation between regret and need for cognition (r = -0.317; p = 0.023), however no relationship was observed among females (r = -0.254; p = 0.576); the difference between these two correlations was non-significant (p = 0.617). There was no association between objectivism and regret among males (r = -0.282; p = 0.098), or among females (r = -0.174; p = 1.00). Likewise, there was no correlation between ambiguity intolerance and regret among males (r = 0.192; p = 1.00) and females (r = 0.276; p = 0.273). There was no significant association between regret and cognitive reflection among males (r = -0.138; p = 1.00) and females (r = 0.137; p = 1.00).

#### Specialty (cognitive v. procedural discipline)

57 participants practiced in a surgical discipline, while 164 participants were non-surgical (cognitive) specialists. There was no relationship between regret and satisficing tendency among non-surgical specialists (r = -0.155; p = 1.00) as well as surgical specialists (r = -0.168; p = 1.00). There was a strong, positive relationship between decision difficulty and regret among both cognitive specialists (r = 0.694; p<0.001) and surgical specialists (r = 0.597; p<0.001). There was no relationship, however, between regret and alternate search among both cognitive specialists (r = 0.219; p = 0.266) and surgeons (r = 0.318; p = 0.884). Both subgroups demonstrated a negative correlation between rational-analytical thinking and regret (non-surgical specialists: r = -0.318; p = 0.002)(surgical specialists: r = -0.519; p = 0.002). No relationship was observed between rational-experiential thinking and regret among both cognitive specialists (r = -0.100; p = 1.00) and surgical specialists (r = -0.255; p = 1.00). Interestingly, non-surgical specialists exhibited no relationship between need for cognition and regret (r = -0.227; p = 0.189), whereas surgical specialists showed a negative one (r = -0.442; p = 0.031); however this difference was not significant (p = 0.121). There was no correlation between regret and objectivism among both non-surgical specialists (r = -0.193; p = 0.725) and surgical specialists (r = -0.354; p = 0.382). There was also no association between regret and ambiguity intolerance among both non-surgical specialists (r = 0.221; p = 0.244) and surgical specialists (r = 0.270; p = 1.00). No significant relationship between regret and cognitive reflection was observed among non-surgical specialists (r = 0.012; p = 1.00) and surgical specialists (r = -0.043; p = 1.00).

### Multivariate Regression Analysis

As demonstrated in Table 2, after bivariate correlational analysis, regret correlated with 6 dimensions of cognitive styles. Table 2 also shows that different cognitive styles intercorrelated with each other. To assess the unique role played by each cognitive style variable as a predictor of regret, we performed a multivariate regression analysis with regret as the dependent variable. The results are shown in Table 3. The F-test indicates that the overall model is statistically significant, thus allowing us to reject the null hypothesis of no associations between regret and its predictors. The strongest predictor of regret was decision difficulty within the maximizing inventory (r = 0.791; p<0.001); however we also observed a statistically significant association with satisficing tendency (r = -0.257; p = 0.020) and objectivism (r = -0.267; p = 0.034) as independent negative predictors of regret. As shown in Table 3, neither the demographic characteristics nor other variables found to be statistically significant in the bivariate analysis remained significant in the multivariate analysis. We can see that approximately 53.9% of the variance can be explained by the statistical model (R^2^ = 0.539). As noted, only the three statistically significant variables (satisficing, decision difficulty, and objectivism) account for the calculated R^2^.

**Table 2:** Means, Standard Deviations (SD), and Intercorrelations of the Scales That Measure Individual Differences in Cognitive Styles & Regret (N = 221).

Scale	Mean	SD	1	2	3	4	5	6	7	8	9
1. MI: Decision Difficulty	3.2	0.758									
2. MI: Alternative Search	3.925	0.821	***0*.*415***								
3. MI: Satisficing	4.86	0.49	0.042	0.18							
4. REI: Rational	2.98	0.531	***-0*.*233***	0.021	**0.226**						
5. REI: Experiential	2.294	0.577	-0.07	0.105	0.198	0.132					
6. Intolerance of Ambiguity	3.068	0.48	0.198	0.194	**-0.216**	***-0*.*346***	-0.141				
7. Need for Cognition	4.241	0.695	-0.172	0.007	0.154	***0*.*745***	0.145	***-0*.*528***			
8. Objectivism	2.766	0.492	-0.076	***0*.*279***	0.154	***0*.*535***	-0.081	-0.02	***0*.*358***		
9. Cognitive Reflection Task	1.49	1.003	-0.091	-0.088	0.08	0.104	0.042	-0.115	0.107	0.006	
10. Regret	2.45	0.99	***0*.*673***	***0*.*239***	-0.156*	***-0*.*366***	-0.141	***0*.*235***	***-0*.*281***	***-0*.*23***	-0.06

Note: MI = Maximizing Inventory; REI = Rational-Experiential Inventory.

Correlation is ***significant*** at the 0.01 level (2-tailed).

Correlation is **significant** at the 0.05 level (2-tailed).

Scale dimensions (higher numbers indicate more of an attribute): MI-Decision Difficulty (1 to 6); MI-Alternative Search (1 to 6); MI-Satisficing (1 to 6); REI-Rational (0 to 4); REI-Experiential (0 to 4); Intolerance of Ambiguity (1 to 6); Need for Cognition (1 to 6); Objectivism (1 to 5); Cognitive Reflection Task (0 to 3); Regret.

*Bonferroni adjustment was applied to the correlational analysis. The correlation between regret and satisficing tendency without the Bonferroni correction is significant at p = 0.0205.

**Table 3:** Linear Multivariate Regression Analysis (Dependent Variable: Regret).

Independent Variable	Regression Coefficient	Standard Error	*P*-value	Standardized Regression Coefficient
MI: Satisficing	-0.257	0.110	0.020	-0.129
MI: Decision Difficulty	0.791	0.071	<0.001	0.617
MI: Alternative Search	0.071	0.071	0.313	0.060
REI: Rational	-0.142	0.153	0.355	-0.077
REI: Experiential	-0.116	0.086	0.180	-0.070
Need for Cognition	-0.040	0.115	0.731	-0.029
Objectivism	-0.267	0.125	0.034	-0.136
Intolerance of Ambiguity	0.050	0.126	0.692	0.025
Cognitive Reflection Task	0.062	0.049	0.201	0.064
Age	-0.012	0.013	0.371	-0.109
Gender	-0.099	0.098	0.315	-0.051
Training Level^1^	0.087	0.145	0.548	0.039
Years of Experience	0.009	0.017	0.572	0.065
Specialty^2^	-0.126	0.107	0.242	-0.057
Constant (Y-intercept)	2.639	1.046	0.212	—-
Number of observations = 218				
F statistics* = 16.96				
Prob > F < 0.001				
R^2^ = 0.539				
Adjusted R^2^ = 0.507				

Note: MI = Maximizing Inventory; REI = Rational-Experiential Inventory.

^1^Training Level: Trainees (residents and fellows) vs. attending physicians

^2^Specialty: Surgical vs. non-surgical disciplines.

## Discussion

Physicians make numerous decisions daily that have substantial consequences for their patients. Many factors influence these decisions, including–but not limited to–clinical experience and expertise, research evidence availability, patient preferences and values, and the individual reasoning style of the clinician. Therefore, medical decision-making is comprised of both an analytical-empirical, as well as an affective-emotional component. One of the most influential emotions on decision-making is regret, which has not been extensively studied in physicians–particularly its relationship with other cognitive strategies that clinicians may employ in their daily decision-making. Here we evaluate the relationship between regret and cognitive styles in physicians. By doing so, we can learn more about what influences medical decision-making, and therefore improve upon it as well.

We found only one other study that directly evaluated the relationship between cognitive styles and regret [7]. In their study of the general population (n = 85), where some participants were physicians, Colombo et al. [7] used the Solomon questionnaire to assess preferences toward intuitive vs. deliberative style decision-making. This scale, which does not include measurements of maximizing and satisficing, consists of 9 items, of which two questions relate to regret (“How many times do you regret/not regret your decisions you made during your working day?”; “A good decision-maker is someone who never regrets his/her decisions”). Interestingly, the authors found that medical doctors reported regretting fewer decisions than the general population. They also found that people with the tendency to engage in analytical thinking reported that a “good decision-maker regrets his/her decisions” much more than those with a propensity toward intuitive thinking (12 vs. 0; Pearson contingency coefficient = 0.52;p<0.05). While in our bivariate analysis we found a negative correlation between regret and tendency for analytical thinking (Table 2), we found no such a relationship in the multivariate regression analysis (Table 3). Colombo and colleagues [7] also discussed their findings within a framework of maximizing and satisficing tendencies. Although they did not directly measure maximizing and satisficing, they believed that their results were consistent with the findings by Schwartz [11] who observed that “maximizers” feel more regret than “satisficiers.” Our data are also consistent with these findings. Of note, Schwartz et al. [11,26] do not measure satisficing directly but treated it as an inverse of maximizing. We used the Maximization Inventory scale [15], which treats satisficing as an independent construct of maximizing. Even so, after conducting a multivariate analysis, we found a positive correlation between regret and maximizing (only with respect to the decision difficulty subscale, but not with respect to the alternative search subscale), and a negative correlation with satisficing (see Table 3). Therefore, among physicians, regret was associated with maximizing tendency, likely due to the fact that maximizers survey more alternative options, which in turn leads to more regret [8,9]. As shown in Table 3, the strongest predictor of regret was decision difficulty, demonstrated by our multivariate regression analysis with regret as the dependent variable (r = 0.791; p<0.001). This indicates that physicians feel more regret as they face more choices while making decisions. Interestingly, we also found that objectivism is a negative predictor of regret (r = -0.267;p = 0.034), signifying that the tendency to seek and use empirical data in decision making as promoted by practice of evidence-based medicine may lead to less regret.

Finally, it is important to place our results in the context of the current theories of human cognitions. Although not uniformly agreed upon [27], we think that our findings fit well within the framework of dual processing theories[1,14,24,28–34]. Dual processing theories posit that the processes underlying problem solving and decision-making are governed by so called Type 1 processes (i.e. intuitive, automatic, fast, narrative, experiential, and affect-based) and Type 2 processes (i.e. analytical, slow, verbal, deliberative and capable of supporting formal logical and probabilistic analyses) [1,14]. Regret is a uniquely human emotion (i.e. type 1 process), which involves counterfactual deliberations (i.e. quintessential type 2 processes). In this context, it is important to note that many problem-solving strategies in medicine rely on heuristics–i.e., simple, “rule of thumb” strategies–for making decisions when time is pressing and long deliberations are not possible [35]. These strategies often attempt to minimize loss [36] but are also geared toward efficient, “good enough”, satisficing solutions when expending less effort may actually result in more accurate solutions [35].

Our data demonstrates the relationship among regret, maximizing, satisficing and objectivism, and support the hypothesis [10] that regret can serve as a link between type 1 and type 2 cognitive processes.

Our findings suggest a plausible–and testable–hypothesis of how regret affects physicians’ behavior. This effect does not appear to be related to accuracy of inferences (as we found no relationship between conditional inference tasks and regret). Instead, we hypothesize that the relationship between regret and maximizing may lead to overtesting as an attempt to compensate for high regret when one has to make a decision among abundant choices. Therefore, we can possibly attribute unnecessary health resource utilization to maximizing tendency and regret proneness. However, we also detected a negative correlation between objectivism (tendency to seek evidence-based, empirical information) and regret indicating that physicians prone to practice evidence-based medicine may feel less regret. Of course, when hypothesizing these relationships, we are aware that the “association is not the same as causation” and that it is possible that regret suppresses the need for objectivism and causes one to maximize. This, as stated, is a testable hypothesis; it would be, for example, very interesting to compare the amount of regret between those physicians inclined to practice evidence-based medicine vs. those who rely on more “traditional” ways of clinical practice.

Our proposed hypothesis is also supported by the insight that maximizing-satisficing tendencies may be malleable, and are not as immutable as once thought. Instead, cognitive reasoning styles likely represent innate predispositions that are shaped by external stimuli [37]. This is suggested by our prior research, where we demonstrated that both maximizing and satisficing tendencies decrease with age [3], and is somewhat supported by our current findings whereby experienced physicians are less regret-prone than trainees (observed in the bivariate correlations but not in the multivariate regression analysis). This implies that interventions targeted at modifying regret proneness, and teaching how emotional and behavioral factors influence decision making, alongside objective evidence-based medicine training, can improve patient care when encountering uncertainty.

While there are significant implications for the field of medical decision-making from our findings, our study is not without limitations. First, the study was limited to only one institution. However, we believe that the academic physicians at the University of South Florida are representative of all U.S. physicians, and therefore our results are generalizable, at least to those physicians who practice in the US. Additionally, our study was conducted as a cross-sectional “snap-shot,” which may have affected the availability and education of participants. In other words, a different “snapshot” may yield a different selection of participating physicians, and possibly generate different results. This possibility, however, can only be confirmed or refuted by future studies aiming to reproduce our results. Finally, we have measured regret using simple Likert scale. While previous research has shown that regret is reliably elicited regardless of how it is measured [22], it is theoretically possible that the use of a different instrument for measuring regret would generate different results. However, we think this is unlikely because we and other researchers [4–6,23]have successfully used simple, one-item scales to elicit regret.

We also want to comment on the differences noted between the bivariate correlational analyses and multivariate regression analysis and our use of Bonferroni’s correction. The results of our multivariate regression analysis does not negate the findings observed in the correlational analyses as the lack of associations observed in the multi-regression analysis could be simply due to the lack of power (we ran a multi-regression analyses on 14 variables with a sample size of 221). It is conceivable that the larger sample sizes could detect statistically significant smaller, yet meaningful, effects. We also note that if Bonferroni’s correction is removed, some correlations in the analysis–such as the relationship between satisficing and regret–become statistically significant. The literature is replete with statistical arguments for and against the use of Boneferroni’s correction [38,39]. In general, Bonferroni’s correction is advisable if one wants to protect against false positives (alpha error), when a large number of tests are carried out without strong *a priori* hypotheses, and when a single test of the universal null hypothesis that all tests are not significant is appropriate [40]. Bonferroni’s correction should not be used if one has a strong theoretical rationale for comparison and wants to avoid false negatives (beta error) [40]. Because we used a number of instruments measuring domains whose relationships (except among regret, maximizing and satisficing) were difficult to predict, we *a priori* opted to be more conservative and use Bonferoni’s adjustment for multiple comparisons. However, given the strong theoretical rationale for the relationship among regret, maximizing, and satisficing, we also believe it is appropriate to conduct the analyses without Bonferroni’s adjustment. Future studies should bear this issue in mind.

Despite its limitations, the results and implications of this study have potential to improve medical decision-making, and therefore patient outcomes. One of the most pressing issues in modern medicine is variation in care, which is thought to be directly related to physicians’ decision-making, behavior, and cognitive processes. Therefore, by understanding how these cognitive processes affect variation in decision-making and resource utilization, we can hopefully improve upon clinical practice.

In conclusion, we believe that our findings of how regret proneness influences reasoning styles have important implications for the field of medical decision-making, particularly at residency and fellowship training level. While our analysis necessitate additional, corroborative research, we do believe that studying both the emotional and analytical aspects of physicians’ reasoning will improve the quality of clinical care.

## Supporting Information

S1 Appendix Consent to Study and Study Survey. (DOC)

S1 File Data Set. The complete data set used in this report.
(XLSX)

S1 Table Conditional Inference Model. The four inferences studied using the conditional inference model with examples of invalid, believable, and invalid, believable clinical scenarios.
(DOCX)
