# Supplementary material for: Thinking Styles and Regret in Physicians
Source: PLoS One. 2015 Aug 4;10(8):e0134038. doi: 10.1371/journal.pone.0134038 (PMC4524595; doi:10.1371/journal.pone.0134038)
Supplement: S1 Appendix — (DOC) [file pone.0134038.s001.doc]

**S1 Appendix. Consent to Study and Study Survey.**

**Consent to Study**

**Background:**

            As you know physicians make a variety of important clinical judgments and crucial decisions affecting the health of their patients daily. Over the past several years researchers have worked with physicians to gain understanding of the types of clinical decisions (e.g., diagnosing conditions, prescribing therapies, evaluating progress, etc.) being made and the situational or contextual features (such as time pressure and limited resources) that affect these decisions. To date however, little research has been d*one to assess the*breadth and depth of decision-making styles employed by physicians.

**Purpose of Study:**

            The purpose of this study is to survey physicians using a battery of scales that assess individual differences in decision-making approaches and styles.

**General Information and Plan of the Study:**

            In this study you will be asked to read and respond to several statements that form multi-item scales that measure individual differences in the ways that people approach decision-making in general. Additionally, you will be asked to complete a conditional inference task. These scales and task have all been developed using sound principles of psychometric theory, however, they have not been administered to physicians.

            At the beginning of the survey you will be asked to provide some brief demographic information (your area of specialty, years in practice, your age and your gender). You will not be asked to submit any identifying information. The surveys will remain anonymous. The survey is estimated to take approximately *30 minutes* and the conditional inference task is estimated to take an additional *30 minutes*. For the survey portion, you will be presented with sets of statements referring to decision making; some are specific to medical decisions while others are quite general. For the conditional inference task, you will be presented with a conditional statement and asked to determine its validity.

**Potential Benefits and Risks:**

            There are no direct benefits to participants associated with participation in this research study. Only potential benefits of the study are to society as a whole. There are no additional risks associated with participation in this research study beyond everyday risks encountered by participants.

**Compensation:**

            A *$50* gift card will be provided for the completion of the survey. Once you have completed all survey questions, you will be given a confirmation code and instructions for redeeming your gift card.

**Consent to Participate:**

Your participation is entirely voluntary and you are free to stop at any time. Your alternative to participation in this study is to not participate. This being said, we would appreciate it very much if you would respond to all the statements.

**Contact information**

            If you have any questions about the study, either before or after participating, you may contact Dr. Jason Beckstead at USF Health, phone: (xxx) xxx-xxxx, email:xxxxx@health.usf.edu. or Dr. Benjamin Djulbegovic at USF Health, phone: (xxx) xxx-xxxx, email: xxxxx@health.usf.edu.

*If you agree to participate in this study, please select “I agree” below to be re-directed to the survey. If you do not wish to take part in this study please select “I do not agree” to leave the site.*

| I agree | I do not agree |
| --- | --- |
|  |  |

Thank you for choosing to participate in our study. Please answer the demographics questions below.

Indicate your area of specialization *(please select only one)*

Please specify 'other' area of specialization.

Are you a

Resident

Fellow

Attending

Which year of fellowship are you currently in?

**OR**

Which year of residency are you currently in?

**OR**

How many years have you practiced in your field?

What is your age?

What is your gender?

|  | Male |  | Female |
| --- | --- | --- | --- |

Please answer the following three questions without any external resources (e.g. internet search, books, etc.).

**OR**

Please answer the following three questions. Feel free to use any external resources (e.g. internet search, books, etc.).

Your hospital has purchased a blood gas analyzer and an MRI scanner for *$2.1 million*. An MRI scanner costs *$2 million* more than the blood gas analyzer. How much does the blood gas analyzer cost?

*Please enter your answer in dollars.*

If it takes*5 surgeons 5 hours* to operate on*5 patients*, how long will it take*20 surgeons to operate*on*20 patients*?

## *Please enter your answer in hours.*

## In a patient with leukemia, the white blood cell count doubles every day.  If it takes 50 days for the white blood cell count to reach 46,000/ccu, how long does it take for the count to reach 23,000/ccu?

## *Please enter your answer in days.*

**Study Survey**

The purpose of this portion of the study is to examine deductive reasoning as applied to medical statements. You will be presented with a number of logical arguments. In each case, you will be asked if a conclusion logically follows from the premises. You should answer each question on the assumption that the information given in the statement is, in fact, true. Answer the questions only on the basis of conclusions that follow logically from the original statement. If the conclusion necessarily follows from the statements, you should answer ‘yes’; otherwise, answer ‘no’.

Please do not use any external resources (e.g. internet search, books, etc.) to complete this portion of the survey.

**OR**

Please feel free to use any external resources (e.g. internet search, books, etc.) to complete this portion of the survey.

*Assume the following is true:*

**If a patient has pulmonary embolism, then the patient is short of breath**

*Given that the following premise is also true:*

**Mrs. Smith has pulmonary embolism**

*Is it logically necessary that:*

**Mrs. Smith is short of breath**

| YES | NO |
| --- | --- |
|  |  |

*Assume the following is true:*

**If a patient has pulmonary embolism, then the patient is short of breath**

*Given that the following premise is also true:*

**Mrs. Smith does not have pulmonary embolism**

*Is it logically necessary that:*

**Mrs. Smith is not short of breath**

| YES | NO |
| --- | --- |
|  |  |

*Assume the following is true:*

**If a patient has pulmonary embolism, then the patient is short of breath**

*Given that the following premise is also true:*

**Mrs. Smith is short of breath**

*Is it logically necessary that:*

**Mrs. Smith has pulmonary embolism**

| YES | NO |
| --- | --- |
|  |  |

*Assume the following is true:*

**If a patient has pulmonary embolism, then the patient is short of breath**

*Given that the following premise is also true:*

**Mrs. Smith is not short of breath**

*Is it logically necessary that:*

**Mrs. Smith does not have pulmonary embolism**

| YES | NO |
| --- | --- |
|  |  |

*Assume the following is true:*

**If a patient has meningitis, then the patient has a stiff neck**

*Given that the following premise is also true:*

**Mr. Jones has meningitis**

*Is it logically necessary that:*

**Mr. Jones has a stiff neck**

| YES | NO |
| --- | --- |
|  |  |

*Assume the following is true:*

**If a patient has meningitis, then the patient has a stiff neck**

*Given that the following premise is also true:*

**Mr. Jones does not have meningitis**

*Is it logically necessary that:*

**Mr. Jones does not have a stiff neck**

| YES | NO |
| --- | --- |
|  |  |

*Assume the following is true:*

**If a patient has meningitis, then the patient has a stiff neck**

*Given that the following premise is also true:*

**Mr. Jones has a stiff neck**

*Is it logically necessary that:*

**Mr. Jones has meningitis**

| YES | NO |
| --- | --- |
|  |  |

*Assume the following is true:*

**If a patient has meningitis, then the patient has a stiff neck**

*Given that the following premise is also true:*

**Mr. Jones does not have a stiff neck**

*Is it logically necessary that:*

**Mr. Jones does not have meningitis**

| YES | NO |
| --- | --- |
|  |  |

*Assume the following is true:*

**If a healthcare worker suffers a needle stick, then an HIV screening test is administered to the worker**

*Given that the following premise is also true:*

**Dr. Collins suffered a needle stick**

*Is it logically necessary that:*

**An HIV screening test is administered to Dr. Collins**

| YES | NO |
| --- | --- |
|  |  |

*Assume the following is true:*

**If a healthcare worker suffers a needle stick, then an HIV screening test is administered to the worker**

*Given that the following premise is also true:*

**Dr. Collins did not suffer a needle stick**

*Is it logically necessary that:*

**An HIV screening test is not administered to Dr. Collins**

| YES | NO |
| --- | --- |
|  |  |

*Assume the following is true:*

**If a healthcare worker suffers a needle stick, then an HIV screening test is administered to the worker**

*Given that the following premise is also true:*

**An HIV screening test is administered to Dr. Collins**

*Is it logically necessary that:*

**Dr. Collins suffered a needle stick**

| YES | NO |
| --- | --- |
|  |  |

*Assume the following is true:*

**If a healthcare worker suffers a needle stick, then an HIV screening test is administered to the worker**

*Given that the following premise is also true:*

**An HIV screening test is not administered to Dr. Collins**

*Is it logically necessary that:*

**Dr. Collins did not suffer a needle stick**

| YES | NO |
| --- | --- |
|  |  |

*Assume the following is true:*

**If a woman is over *55 years* old, then the woman benefits from mammography for breast cancer screening**

*Given that the following premise is also true:*

**Mrs. Kelli is over *55 years* old**

*Is it logically necessary that:*

**Mrs. Kelli benefits from mammography for breast cancer screening**

| YES | NO |
| --- | --- |
|  |  |

*Assume the following is true:*

**If a woman is over *55 years* old, then the woman benefits from mammography for breast cancer screening**

*Given that the following premise is also true:*

**Mrs. Kelli is not over *55 years* old**

*Is it logically necessary that:*

**Mrs. Kelli does not benefit from mammography for breast cancer screening**

| YES | NO |
| --- | --- |
|  |  |

*Assume the following is true:*

**If a woman is over *55 years* old, then the woman benefits from mammography for breast cancer screening**

*Given that the following premise is also true:*

**Mrs. Kelli benefits from mammography for breast cancer screening**

*Is it logically necessary that:*

**Mrs. Kelli is over *55 years* old**

| YES | NO |
| --- | --- |
|  |  |

*Assume the following is true:*

**If a woman is over *55 years* old, then the woman benefits from mammography for breast cancer screening**

*Given that the following premise is also true:*

**Mrs. Kelli does not benefit from mammography for breast cancer screening**

*Is it logically necessary that:*

**Mrs. Kelli is not over *55 years* old**

| YES | NO |
| --- | --- |
|  |  |

*Assume the following is true:*

**If a patient has appendicitis, then the patient is treated with antibiotics**

*Given that the following premise is also true:*

**Mr. Thomas has appendicitis**

*Is it logically necessary that:*

**Mr. Thomas is treated with antibiotics**

| YES | NO |
| --- | --- |
|  |  |

*Assume the following is true:*

**If a patient has appendicitis, then the patient is treated with antibiotics**

*Given that the following premise is also true:*

**Mr. Thomas does not have appendicitis**

*Is it logically necessary that:*

**Mr. Thomas is not treated with antibiotics**

| YES | NO |
| --- | --- |
|  |  |

*Assume the following is true:*

**If a patient has appendicitis, then the patient is treated with antibiotics**

*Given that the following premise is also true:*

**Mr. Thomas is treated with antibiotics**

*Is it logically necessary that:*

**Mr. Thomas has appendicitis**

| YES | NO |
| --- | --- |
|  |  |

*Assume the following is true:*

**If a patient has appendicitis, then the patient is treated with antibiotics**

*Given that the following premise is also true:*

**Mr. Thomas is not treated with antibiotics**

*Is it logically necessary that:*

**Mr. Thomas does not have appendicitis**

| YES | NO |
| --- | --- |
|  |  |

*Assume the following is true:*

**If a dying patient has a do not resuscitate (DNR) order, then the patient is allowed to die**

*Given that the following premise is also true:*

**Ms. Morris, a dying patient, has a do not resuscitate (DNR) order**

*Is it logically necessary that:*

**Ms. Morris is allowed to die**

| YES | NO |
| --- | --- |
|  |  |

*Assume the following is true:*

**If a dying patient has a do not resuscitate (DNR) order, then the patient is allowed to die**

*Given that the following premise is also true:*

**Ms. Morris, a dying patient, does not have a do not resuscitate (DNR) order**

*Is it logically necessary that:*

**Ms. Morris is not allowed to die**

| YES | NO |
| --- | --- |
|  |  |

*Assume the following is true:*

**If a dying patient has a do not resuscitate (DNR) order, then the patient is allowed to die**

*Given that the following premise is also true:*

**Ms. Morris is allowed to die**

*Is it logically necessary that:*

**Ms. Morris, a dying patient, has a do not resuscitate (DNR) order**

| YES | NO |
| --- | --- |
|  |  |

*Assume the following is true:*

**If a dying patient has a do not resuscitate (DNR) order, then the patient is allowed to die**

*Given that the following premise is also true:*

**Ms. Morris is not allowed to die**

*Is it logically necessary that:*

**Ms. Morris, a dying patient, does not have a do not resuscitate (DNR) order**

| YES | NO |
| --- | --- |
|  |  |

*Assume the following is true:*

**If a patient presents with confusion, then the patient has a brain tumor**

*Given that the following premise is also true:*

**Mr. Hartford presents with confusion**

*Is it logically necessary that:*

**Mr. Hartford has a brain tumor**

| YES | NO |
| --- | --- |
|  |  |

*Assume the following is true:*

**If a patient presents with confusion, then the patient has a brain tumor**

*Given that the following premise is also true:*

**Mr. Hartford does not present with confusion**

*Is it logically necessary that:*

**Mr. Hartford does not have a brain tumor**

| YES | NO |
| --- | --- |
|  |  |

*Assume the following is true:*

**If a patient presents with confusion, then the patient has a brain tumor**

*Given that the following premise is also true:*

**Mr. Hartford has a brain tumor**

*Is it logically necessary that:*

**Mr. Hartford presents with confusion**

| YES | NO |
| --- | --- |
|  |  |

*Assume the following is true:*

**If a patient presents with confusion, then the patient has a brain tumor**

*Given that the following premise is also true:*

**Mr. Hartford does not have a brain tumor**

*Is it logically necessary that:*

**Mr. Hartford does not present with confusion**

| YES | NO |
| --- | --- |
|  |  |

*Assume the following is true:*

**If a patient has a high fever, then the patient has malaria**

*Given that the following premise is also true:*

**Ms. Boyle has a high fever**

*Is it logically necessary that:*

**Ms. Boyle has malaria**

| YES | NO |
| --- | --- |
|  |  |

*Assume the following is true:*

**If a patient has a high fever, then the patient has malaria**

*Given that the following premise is also true:*

**Ms. Boyle does not have a high fever**

*Is it logically necessary that:*

**Ms. Boyle does not have malaria**

| YES | NO |
| --- | --- |
|  |  |

*Assume the following is true:*

**If a patient has a high fever, then the patient has malaria**

*Given that the following premise is also true:*

**Ms. Boyle has malaria**

*Is it logically necessary that:*

**Ms. Boyle has a high fever**

| YES | NO |
| --- | --- |
|  |  |

*Assume the following is true:*

**If a patient has a high fever, then the patient has malaria**

*Given that the following premise is also true:*

**Ms. Boyle does not have malaria**

*Is it logically necessary that:*

**Ms. Boyle does not have a high fever**

| YES | NO |
| --- | --- |
|  |  |

*Assume the following is true:*

**If a patient has a sore throat, then the patient has cancer of the esophagus**

*Given that the following premise is also true:*

**Mr. Fisher has a sore throat**

*Is it logically necessary that:*

**Mr. Fisher has cancer of the esophagus**

| YES | NO |
| --- | --- |
|  |  |

*Assume the following is true:*

**If a patient has a sore throat, then the patient has cancer of the esophagus**

*Given that the following premise is also true:*

**Mr. Fisher does not have a sore throat**

*Is it logically necessary that:*

**Mr. Fisher does not have cancer of the esophagus**

| YES | NO |
| --- | --- |
|  |  |

*Assume the following is true:*

**If a patient has a sore throat, then the patient has cancer of the esophagus**

*Given that the following premise is also true:*

**Mr. Fisher has cancer of the esophagus**

*Is it logically necessary that:*

**Mr. Fisher has a sore throat**

| YES | NO |
| --- | --- |
|  |  |

*Assume the following is true:*

**If a patient has a sore throat, then the patient has cancer of the esophagus**

*Given that the following premise is also true:*

**Mr. Fisher does not have cancer of the esophagus**

*Is it logically necessary that:*

**Mr. Fisher does not have a sore throat**

| YES | NO |
| --- | --- |
|  |  |

*Assume the following is true:*

**If a patient is overw*eight by*10% over his normal body weight, then he will die next year**

*Given that the following premise is also true:*

**Mr. Klein is overw*eight by*10% over his normal body weight**

*Is it logically necessary that:*

**Mr. Klein will die next year**

| YES | NO |
| --- | --- |
|  |  |

*Assume the following is true:*

**If a patient is overw*eight by*10% over his normal body weight, then he will die next year**

*Given that the following premise is also true:*

**Mr. Klein is not overw*eight by*10% over his normal body weight**

*Is it logically necessary that:*

**Mr. Klein will not die next year**

| YES | NO |
| --- | --- |
|  |  |

*Assume the following is true:*

**If a patient is overw*eight by*10% over his normal body weight, then he will die next year**

*Given that the following premise is also true:*

**Mr. Klein will die next year**

*Is it logically necessary that:*

**Mr. Klein is overw*eight by*10% over his normal body weight**

| YES | NO |
| --- | --- |
|  |  |

*Assume the following is true:*

**If a patient is overw*eight by*10% over his normal body weight, then he will die next year**

*Given that the following premise is also true:*

**Mr. Klein will not die next year**

*Is it logically necessary that:*

**Mr. Klein is not overw*eight by*10% over his normal body weight**

| YES | NO |
| --- | --- |
|  |  |

*Assume the following is true:*

**If ECG of hospitalized patients is regularly monitored, then all deaths in US hospitals will be prevented**

*Given that the following premise is also true:*

**Tampa General Hospital regularly monitors ECG of hospitalized patients**

*Is it logically necessary that:*

**All deaths at Tampa General Hospital will be prevented**

| YES | NO |
| --- | --- |
|  |  |

*Assume the following is true:*

**If ECG of hospitalized patients is regularly monitored, then all deaths in US hospitals will be prevented**

*Given that the following premise is also true:*

**Tampa General Hospital does not regularly monitor ECG of hospitalized patients**

*Is it logically necessary that:*

**All deaths at Tampa General Hospital will not be prevented**

| YES | NO |
| --- | --- |
|  |  |

*Assume the following is true:*

**If ECG of hospitalized patients is regularly monitored, then all deaths in US hospitals will be prevented**

*Given that the following premise is also true:*

**All deaths at Tampa General Hospital will be prevented**

*Is it logically necessary that:*

**Tampa General Hospital regularly monitors ECG of hospitalized patients**

| YES | NO |
| --- | --- |
|  |  |

*Assume the following is true:*

**If ECG of hospitalized patients is regularly monitored, then all deaths in US hospitals will be prevented**

*Given that the following premise is also true:*

**All deaths at Tampa General Hospital will not be prevented**

*Is it logically necessary that:*

**Tampa General Hospital does not regularly monitor ECG of hospitalized patients**

| YES | NO |
| --- | --- |
|  |  |

*Assume the following is true:*

**If a*30 year-old patient*is injured during a soccer match, then the patient will require an abdominal surgery**

*Given that the following premise is also true:*

**Ms. Wilkes, a*30 year-old*, is injured during a soccer match**

*Is it logically necessary that:*

**Ms. Wilkes will require an abdominal surgery**

| YES | NO |
| --- | --- |
|  |  |

*Assume the following is true:*

**If a*30 year-old patient*is injured during a soccer match, then the patient will require an abdominal surgery**

*Given that the following premise is also true:*

**Ms. Wilkes, a*30 year-old*, is not injured during a soccer match**

*Is it logically necessary that:*

**Ms. Wilkes will not require an abdominal surgery**

| YES | NO |
| --- | --- |
|  |  |

*Assume the following is true:*

**If a*30 year-old patient*is injured during a soccer match, then the patient will require an abdominal surgery**

*Given that the following premise is also true:*

**Ms. Wilkes will require an abdominal surgery**

*Is it logically necessary that:*

**Ms. Wilkes, a*30 year-old*, is injured during a soccer match**

| YES | NO |
| --- | --- |
|  |  |

*Assume the following is true:*

**If a*30 year-old patient*is injured during a soccer match, then the patient will require an abdominal surgery**

*Given that the following premise is also true:*

**Ms. Wilkes will not require an abdominal surgery**

*Is it logically necessary that:*

**Ms. Wilkes, a*30 year-old*, is not injured during a soccer match**

| YES | NO |
| --- | --- |
|  |  |

Below are several statements describing how people think and feel about decision making. Please read each statement carefully. Indicate the extent to which you agree or disagree with each statement using the following rating scale:

| 1 | 2 | 3 | 4 | 5 | 6 |
| --- | --- | --- | --- | --- | --- |
| Strongly disagree | Disagree | Slightly disagree | Slightly agree | Agree | Strongly agree |

1. ____ I usually try to find a couple of good options and then choose between them.

2. ____ I usually have a hard time making even simple decisions.

3. ____ I can’t come to a decision unless I have carefully considered all of my options.

4. ____ At some point you need to make a decision about things.

5. ____ I am usually worried about making a wrong decision.

6. ____ I take time to read the whole menu when dining out.

7. ____ In life I try to make the most of whatever path I take.

8. ____ I often wonder why decisions can’t be more easy.

9. ____ I will continue shopping for an item until it reaches all of my criteria.

10. ____ There are usually several good options in a decision situation.

11. ____ I often put off making a difficult decision until a deadline.

12. ____ I usually continue to search for an item until it reaches my expectations.

13. ____ I try to gain plenty of information before I make a decision, but then I go ahead and make it.

14. ____ I often experience buyer’s remorse.

15. ____ When shopping, I plan on spending a lot of time looking for something.

16. ____ Good things can happen even when things don’t go right at first.

17. ____ I often think about changing my mind after I have already made my decision.

18. ____ When shopping, if I can’t find exactly what I’m looking for, I will continue to search for it.

19. ____ I can’t possibly know everything before making a decision.

20. ____ The hardest part of making a decision is knowing I will have to leave the item I didn’t choose

behind.

21. ____ I find myself going to many different stores before finding the thing I want.

22. ____ I do not agonize over decisions.

23. ____ I just won’t make a decision until I am comfortable with the process.

24. ____ All decisions have pros and cons.

25. ____ I often change my mind several times before making a decision.

26. ____ When shopping for something, I don’t mind spending several hours looking for it.

27. ____ I know that if I make a mistake in a decision that I can go “back to the drawing board.”

28. ____ It’s hard for me to choose between two good alternatives.

29. ____ I take the time to consider all alternatives before making a decision.

30. ____ I accept that life often has uncertainty.

31. ____ Sometimes I procrastinate in deciding even if I have a good idea of what decision I will make.

32. ____ When I see something that I want, I always try to find the best deal before purchasing it.

33. ____ I find myself often faced with difficult decisions.

34. ____ If a store doesn’t have exactly what I’m shopping for, then I will go somewhere else.

35. ____ I take time to consider all available options when making a diagnosis.

*36*. ____ I often experience regret following a decision.

*37*. ____ I find myself going to many colleagues before finding the answer I want.

*38*. ____ When making a management decision, I don't mind spending a long time doing it.

*39*. ____ If a colleague doesn't have exactly what I need when I'm seeking a second opinion, I will go somewhere else.

Below are several statements that describe how various people make decisions in general. Read each statement carefully and think about the extent to which the statement describes you. Use the following rating scale to indicate your responses.

| 0 | 1 | 2 | 3 | 4 |
| --- | --- | --- | --- | --- |
| Not at all characteristic of me | Slightly characteristic of me | Moderately characteristic of me | Very characteristic of me | Extremely characteristic of me |

1. _____ I would not want to depend on anyone who described himself or herself as intuitive.

2. _____ My snap judgments are probably not as good as most people's.

3. _____ I tend to use my heart as a guide for my actions.

4. _____ I can usually feel when a person is right or wrong, even if I can't explain how I know.

5. _____ I suspect my hunches are inaccurate as often as they are accurate.

6. _____ I try to avoid situations that require thinking in depth about something.

7. _____ I'm not that good at figuring out complicated problems.

8. _____ When it comes to trusting people, I can usually rely on my gut feelings.

9. _____ I enjoy intellectual challenges.

10. _____ I am not very good at solving problems that require careful logical analysis.

11. _____ I don't like to have to do a lot of thinking.

12. _____ I often go by my instincts when deciding on a course of action.

13. _____ I trust my initial feelings about people.

14. _____ If I were to rely on my gut feelings, I would often make mistakes.

15. _____ I don't like situations in which I have to rely on intuition.

16. _____ Knowing the answer without having to understand the reasoning behind it is good enough

for me.

17. _____ I don't reason well under pressure.

18. _____ I am much better at figuring things out logically than most people.

19. _____ I have a logical mind.

20. _____ I enjoy thinking in abstract terms.

21. _____ Thinking hard and for a long time about something gives me little satisfaction.

22. _____ I think there are times when one should rely on one's intuition.

23. _____ I think it is foolish to make important decisions based on feelings.

24. _____ I don't think it is a good idea to rely on one's intuition for important decisions.

25. _____ I generally don't depend on my feelings to help me make decisions.

26. _____ I hardly ever go wrong when I listen to my deepest gut feelings to find an answer.

27. _____ I have no problem thinking things through carefully.

28. _____ Using logic usually works well for me in figuring out problems in my life.

29. _____ I usually have clear, explainable reasons for my decisions.

30. _____ Learning new ways to think would be very appealing to me.

31. _____ I like to rely on my intuitive impressions.

32. _____ I don't have a very good sense of intuition.

33. _____ Using my gut feelings usually works well for me in figuring out problems in my life.

34. _____ I believe in trusting my hunches.

35. _____ Intuition can be a very useful way to solve problems.

36. _____ I enjoy solving problems that require hard thinking.

37. _____ Thinking is not my idea of an enjoyable activity.

38. _____ I am not a very analytical thinker.

39. _____ Reasoning things out carefully is not one of my strong points.

40. _____ I prefer complex problems to simple problems.

Below are several general statements regarding how people perceive and think about the world around them. Please read each statement carefully. Indicate the extent to which you agree or disagree with each statement using the following rating scale:

| 1 | 2 | 3 | 4 | 5 | 6 |
| --- | --- | --- | --- | --- | --- |
| Strongly disagree | Disagree | Slightly disagree | Slightly agree | Agree | Strongly agree |

1. _____ An expert who doesn't come up with a definite answer probably doesn't know very much.

2. _____ Teachers or supervisors who hand out vague assignments give a chance for one to show

initiative and originality.

3. _____ People who fit their lives to a schedule probably miss most of the joy of living.

4. _____ Often the most interesting and stimulating people are those who don't mind being different

and original.

5. _____ It is more fun to tackle a complicated problem than to solve a simple one.

6. _____ In the long run it is possible to get more done by tackling small, simple problems rather

than large and complicated ones.

7. _____ A good job is one where what is to be done and how it is to be done are always clear.

8. _____ A person who leads an even, regular life in which few surprises or unexpected happenings

arise, really has a lot to be grateful for.

9. _____ What we are used to is always preferable to what is unfamiliar to us.

10. _____ People who insist upon a "yes" or "no" answer just don't know how complicated things

really are.

11. _____ There is really no such thing as a problem that can't be solved.

12. _____ Many of our most important decisions are based upon insufficient information.

13. _____ I like parties where I know most of the people more than ones where all or most of the

people are complete strangers.

14. _____ I would like to live in a foreign country for a while.

15. _____ The sooner we all acquire similar values and ideals the better.

16. _____ A good teacher is one who makes you wonder about your own way of looking at things.

Below are several statements about thinking and problem solving. Please read each statement carefully and choose the numbered response that best describes your opinion.

| 1 | 2 | 3 | 4 | 5 | 6 |
| --- | --- | --- | --- | --- | --- |
| Strongly disagree | Disagree | Slightly disagree | Slightly agree | Agree | Strongly agree |

1. ____ I prefer complex to simple problems.

2. ____ I like to have the responsibility of handling a situation that requires a lot of thinking.

3. ____ Thinking is not my idea of fun.

4. ____ I'd rather do something requiring little thought than something sure to challenge my

thinking abilities.

5. ____ I try to anticipate and avoid situations where I may have to think in depth about

something.

6. ____ I find satisfaction in deliberating hard and for long hours.

7. ____ I only think as hard as I have to.

8. ____ I prefer to think about small, daily projects compared to long-term ones.

9. ____ I like tasks that require little thought once I've learned them.

10.____ The idea of relying on thought to make my way to the top appeals to me.

11. ____ I really enjoy a task that involves coming up with new solutions to problems.

12. ____ Learning new ways to think doesn't excite me very much.

13. ____ I prefer my life to be filled with puzzles that I must solve.

14. ____ The notion of thinking abstractly is appealing to me.

15. ____ I would prefer a task that is intellectual, difficult, and important to one that's somewhat

important but doesn't require much thought.

16. ____ I feel relief rather than satisfaction after completing a task that required a lot of

mental effort.

17. ____ It's enough for me that something gets the job done; I don't care how or why it works.

Below are several statements that describe how various people make decisions in general. Read each statement carefully and think about the extent to which the statement describes you. Use the following rating scale to indicate your responses.

| 0 | 1 | 2 | 3 | 4 |
| --- | --- | --- | --- | --- |
| Not at all characteristic of me | Slightly characteristic of me | Moderately characteristic of me | Very characteristic of me | Extremely characteristic of me |

1. ____ I seek as much information as possible before making decisions.

2. ____ I think the answers to most questions in life can be found through careful, objective

analysis of the situation.

3. ____ I do not like to be too objective in the way I look at things.

4. ____ Trying to be highly objective and rational does not improve my ability to make good

decisions.

5. ____ I see myself as a rational and objective person.

6. ____ After I make a decision, it is often difficult for me to give logical reasons for it.

7. ____ I gather as much information as possible before making decisions.

8. ____ The solution to many problems in life cannot be found through an intellectual

examination of the facts.

9. ____ I try to employ a cool-headed, objective approach when making decisions about my life.

10. ____ I am only confident of decisions that are made after careful analysis of all available

information.

11. ____ I tend not to be particularly objective or logical in my approach to life.
