# Supplementary material for: Thinking Styles and Regret in Physicians
Source: PLoS One. 2015 Aug 4;10(8):e0134038. doi: 10.1371/journal.pone.0134038 (PMC4524595; doi:10.1371/journal.pone.0134038)
Supplement: S1 Table — The four inferences studied using the conditional inference model with examples of invalid, believable, and invalid, believable clinical scenarios. (DOCX) [file pone.0134038.s003.docx]

| **Inference** | **Clinical Scenario Example** | **Major premise** | **Minor premise** | **Conclusion** | **Validity** |
| --- | --- | --- | --- | --- | --- |
| Modus ponens (MP) | Unbelievable example:  *Assume the following is true:*  ***If a patient has a high fever, then the patient has malaria.***  *Given that the following premise is also true:*  ***Ms. Boyle has a high fever.***  *Is it necessary that:*  ***Ms. Boyle has malaria.***  *○* ***YES*** *○* ***NO*** | If A then B | A | B | VALID |
| Denial of antecedent (DA) | Believable example:  *Assume the following is true:*  ***If a patient has pulmonary embolism, then the patient is short of breath.***  *Given that the following premise is also true:*  ***Mrs. Smith does not have pulmonary embolism.***  *Is it necessary that:*  ***Mrs. Smith is not short of breath.***  *○* ***YES*** *○* ***NO*** | If A then B | not A | not B | INVALID |
| Affirmation of the consequent (AC) | Believable example:  *Assume the following is true:*  ***If a patient has pulmonary embolism, then the patient is short of breath.***  Given that the following premise is also true:  ***Mrs. Smith is short of breath.***  Is it necessary that:  ***Mrs. Smith has pulmonary embolism.***  ***○ YES ○ NO*** | If A then B | B | A | INVALID |
| Modus tollens (MT) | Unbelievable example:  *Assume the following is true:*  ***If a patient has a high fever, then the patient has malaria.***  Given that the following premise is also true:  ***Ms. Boyle does not have malaria.***  Is it necessary that:  ***Ms. Boyle does not have a high fever.***  ***○ YES ○ NO*** | If A then B | not B | not A | VALID |

**S1 Table. Conditional Inference Model.** The four inferences studied using the conditional inference model with examples of invalid, believable, and invalid, unbelievable clinical scenarios
